# Ethnicity, Stigma and Adherence to Antiretroviral Therapy (ART) among People Living with HIV/AIDS in Guangxi, China

**DOI:** 10.4172/2155-6113.1000652

**Published:** 2017-01-12

**Authors:** Yuchen Mao, Xiaoming Li, Shan Qiao, Yuejiao Zhou, Qun Zhao

**Affiliations:** Department of Health Promotion, Education and Behavior, University of South Carolina, Columbia, USA

**Keywords:** Ethnicity, Stigma, Adherence, Antiretroviral therapy, People living with HIV/AIDS

## Abstract

This study examines the impact of ethnicity and multiple types of HIV-related stigma on adherence to antiretroviral therapy (ART) among 2,146 people living with HIV/AIDS (PLWHA) in Guangxi, China who had initiated ART. The results of multiple binary logistic regressions indicate that those who had experienced enacted stigma tended to report lower adherence, while better adherence was associated with older age, being women and having a job. Ethnicity had a moderator effect on the association between internalized stigma and adherence since better adherence was associated with lower internalized stigma among participants in ethnic minority groups other than Zhuang. Our findings indicate that PLWHA of other ethnic minority groups could benefit from internalized stigma reduction interventions; PLWHA, overall, could benefit most from increased employment opportunities and acquisition of coping skills to mitigate the negative effects of enacted stigma.

## Introduction

In China, although people living with HIV/AIDS (PLWHA) only accounted for 0.037% of the total population by the end of 2014, the prevalence is higher in some areas including Guangxi Autonomous Region (Guangxi). Meanwhile, the number of patients receiving antiretroviral therapy (ART) has been steadily increasing by a large margin each year. The percentage of PLWHA receiving ART increased from 52.1% in 2013 to 59.0% in 2014 and the percentage of PLWHA with CD4 counts ≤ 350/mm^3^ receiving ART increased from 81.9% in 2011 to 86.9% in 2014 (Ministry of Health China, 2015).

Most HIV patients have to receive lifelong ART, adherence to which is the practice of taking medications consistent with the prescribed treatment regimen, at the correct time interval and with the exact dose [1,2]. High levels of adherence are necessary to avoid development of resistant viruses and in turn abate HIV disease progression [3,4]. Many factors are associated with low adherence or non-adherence and have been documented by various studies [5–9]. For example, Genberg et al. [8] categorized barriers to ART adherence into 4 groups: medication and health concerns, stigma, family responsibilities and problems with schedule and routine. Okoror et al. [1] identified seeking alternative or traditional care, financial cost related to transportation among other things, side effects of medication, and stigma of being identified as HIV positive as factors for nonadherence. It is evident that HIV-related stigma has often been identified as one of the major barriers to adherence.

Some studies have focused specifically on the association of adherence to ART with stigma [1,10–12]. The findings, almost consistently, were that higher levels of stigma could result in lower levels of adherence. Some studies attempted to ascertain the associations of adherence with different types of stigma, including enacted, perceived and internalized stigma [13–16]. For example, Lyimo et al. [17] identified that perceived stigma compared to enact and internalized stigma could have greater impact on disclosure of HIV status which in turn affects adherence. However, the associations of different types of stigma with adherence are not yet well understood, especially in the resource-limited settings.

Ethnic affiliation can reflect cultural standards, social policies, and social structure pertinent to HIV [18]. Prior studies have identified that racial and ethnic minorities tended to have higher levels of stigma [19–22] and lower levels of adherence to ART [23–25]. For example, Kunstadter et al. [21] indicated that Yunnan Chinese as an ethnic minority group in north-western Thailand had the lowest level of HIV-related knowledge compared to other ethnic groups, which could be associated with high stigma, low adherence and poor health outcomes. Shih [22] showed that ethnic minorities due to ethnic marginalization were labelled as being at high risk for HIV infection by the Chinese Center for Disease Control’s public campaigns; thus their HIV-related stigma was exacerbated, and their adherence to HIV treatment was affected to a greater extent compared to the ethnic majority. The associations of ethnicity with stigma and adherence are still poorly understood in China, notwithstanding.

To date, the adherence to ART is suboptimal in China [26–28]. For example, in a study conducted among HIV-infected adults in Guangzhou, the percentage of participants who reported recent nonadherence was nearly 19% [27]. Meanwhile, some previous studies indicated that HIV-related stigma could be higher in Asian countries including China [29–32]. Understanding how ethnicity, HIV-related stigma and adherence to ART interact with each other is important for more targeted interventions to improve adherence to ART and in turn health outcomes. Given that few studies have simultaneously focused on the roles of ethnicity and stigma in adherence to ART, the current study aims to examine the impact of ethnicity and multiple types of HIV-related stigma on adherence to ART among PLWHA in Guangxi, China, which is relatively resource-limited compared to the economically-developed provinces in China. We hypothesize that there is a synergistic effect between HIV-related stigma and ethnic minority status on adherence.

## Methods

### Study participants

We conducted a cross-sectional study from 2012 to 2013 in Guangxi in Southwest China. Guangxi was ranked third among Chinese provinces in terms of HIV seropositive cases by the end of 2014 (Ministry of Health of China, 2015). With the assistance and collaboration of Guangxi Center for Disease Prevention and Control (Guangxi CDC), we randomly selected PLWHA from the top 12 sites (2 cities and 10 counties) with the largest number of HIV/AIDS cases (about 10% of the reported cases at each site were selected).

The detailed sampling and survey procedures were reported elsewhere [33]. Briefly, the self-administered survey was conducted in offices of local CDC or HIV clinics where the participants received medical care. The interviewers were local CDC staff or health care workers in the HIV clinics who had received intensive training on research ethics and interview skills with PLWHA prior to the field data collection. The Institutional Review Boards at Wayne State University in the United States and Guangxi CDC in China reviewed and approved the research protocol. Among the 2,987 PLWHA completing the survey, a sample of 2,146 participants who had initiated ART was included in the current study.

## Measures

### Background characteristics

Participants were asked about individual and family characteristics including gender, age, ethnicity, marital status, place of original residence (local vs non-local), year of schooling, work status (full-time job, part-time job and no job) and monthly household income in Chinese Yuan (<1,000, 1,000–1,999, 2,000–2,999, 3,000–3,999, 4,000–4,999, and ≥ 5,000). For data analysis in the current study, we categorized ethnicity into three groups: Han, Zhuang and Other. We also dichotomized marital status into married/cohabitating and not married/cohabitating and work status into having a full-time or part-time job and having no job. In addition, we combined the three high-level income categories 3,000–3,999, 4,000–4,999 and ≥ 5,000 into one group (“≥ 3,000”) because of the relatively small number of respondents within each of these categories.

### HIV-related stigma

HIV-related stigma was measured using modified items from the Berger HIV Stigma Scale that was previously validated in various studies [34]. Sixteen modified items were used to quantify the multiple domains of HIV-related stigma including enacted, perceived, and internalized stigma. Enacted stigma was measured using 2 items which asked participants whether they had experienced some stigmatized action (i.e., “I was denied jobs, school or social welfare because of HIV status”, “My family members were excluded by others because of my HIV status”). If participants indicated they had experienced either of the stigmatized actions, their enacted stigma score was coded as “1” (Yes). Otherwise, their enacted stigma score was coded as “0” (No). Perceived stigma was measured using 6 items (e.g. “People with HIV/AIDS lose their jobs when their employers find out”, “Most people believe a person with HIV/AIDS is disgusting”) and internalized stigma was measured using 8 items (e.g. “I feel guilty because I have HIV”, “Having HIV makes me feel that I am bad”). Responses for both perceived and internalized stigma items ranged from 1 (Strongly Disagree) to 4 (Strongly Agree). Reliability was satisfactory with Cronbach’s α of 0.90 and 0.91 for perceived and internalized stigma, respectively.

### Adherence

Four questions from the AIDS Clinical Trials Group Adherence Questionnaire were revised to gather information on adherence to ART in the current study [35]. Participants were asked whether they missed doses over the past 3 days, over the most recent weekend, and over the past month. The responses were then converted into percentage of prescribed doses and recoded into 1 (≥ 90% of prescribed doses) or 0 (<90%). Whether participants ever missed a dose previously was also assessed and coded into 1 (yes) or 0 (no). An overall adherence score was calculated by summing scores of the four adherence behaviors. For the purpose of analysis, we dichotomized participants based on their overall adherence score into optimal (scored 4) and suboptimal adherence groups (scored less than 4).

### Data analysis

Statistical analyses were conducted using SAS 9.4 (Statistical Analysis Software, Cary, NC). We first evaluated all types of stigma and the key socio-demographic characteristics in bivariate analyses for their associations with adherence. Specifically, single-predictor binary logistic regressions were conducted to examine the bivariate association between each potential predictor and adherence. Because the participants were nested in 12 sites, generalized estimating equation (GEE) approach was used to account for intraclass correlation among participants from the same site and performed using SAS GENMOD procedure with a “binomial” distribution, a “logit” link function and an “exchangeable” correlation structure. Thereafter, three multiple binary logistic regression models with the above-mentioned GEE approach were executed. The first model was designed to examine the main effects of ethnicity and multiple types of stigma on adherence. The second model was designed to examine how ethnicity and different types of stigma were related to adherence while controlling for key socio-demographic characteristics. The third model tested the hypothesized interaction of multiple types of stigma with ethnicity in forecasting the treatment adherence over and beyond the previous models.

## Results and Discussion

### Sample characteristics

The sample in the current analysis include 2,146 participants (1,316 males and 830 females) who reported being on ART at the time of survey. A majority of the participants reported a good medication adherence across the four adherence measures (96.6% for adherence based on missing doses over the past 3 days, 96.1% for adherence based on missing doses over the past weekend, 93.3% for adherence based on missing doses over the past month and 66.3% never missed any dose). The sample’s average age and year of schooling were 42.4 years (SD=12.5) and 7.01 (SD=3.02), respectively. More than two-thirds (68.5%) of the participants were of Han ethnicity, a little less than one-third (28.3%) were of Zhuang ethnicity, and those from other ethnic minority groups accounted for less than 5% (3.3%). A majority of the participants were local (93.5%), had a full-time or part-time job (73.6%), had a monthly household income less than 2,000 yuan (82.7%) and reported being married or in cohabitation (69.7%). A majority of the participants (93.9%) did not report experiencing enacted stigma, and on average, participants’ perceived (mean=2.60, SD=0.58) and internalized stigma scores (mean=2.31, SD=0.54) were at the mid-point of the 4-point scale (Table 1).

### Bivariate analysis

Table 1 also summarizes the bivariate associations of adherence with the key sociodemographic characteristics and stigma, which were measured by crude odds ratio (cOR) and its 95% confidence interval. Enacted stigma (cOR=0.73, 95% CI: 0.60, 0.94) tended to be negatively associated with adherence, which indicates that participants who did not experience enacted stigma were more likely to report better adherence. Meanwhile, age (cOR=1.02, 95% CI: 1.01, 1.03) tended to be the only key sociodemographic characteristic associated with adherence, which means that older participants were more likely to have better adherence.

### Binary logistic regressions

The results of the binary logistic regressions were shown in Table 2 and included adjusted odds ratio (aOR) with 95% confidence interval for each predictor in the models. In the sample overall, the main effect of enacted stigma was detected since participants who did not experience enacted stigma tended to report better adherence (aOR=0.66, 95% CI: 0.52, 0.85) (Model 1). Moreover, better adherence was associated with older age (aOR=1.02, 95% CI: 1.01, 1.03), being women (aOR=1.16, 95% CI: 1.05, 1.29), and having a full-time or part-time job (aOR=1.31, 95% CI: 1.02, 1.68) (Model 2). When the multiplicative interaction terms between ethnicity and the three types of stigma were added (Model 3), the analyses revealed that compared to Han and Zhuang ethnic groups, adherence among other ethnic groups tended to be lower due to the existence of internalized stigma and negatively associated with internalized stigma (aOR=0.40, 95% CI: 0.17, 0.96). Therefore, ethnicity was found to have a moderator effect on the association between internalized stigma and adherence.

## Conclusion

The current study examined adherence to ART in relation to multiple types of stigma and ethnicity among PLWHA in Guangxi, China, and identified factors that could influence adherence. The main effect of enacted stigma, age, gender, and work status in adherence were found; meanwhile, ethnicity was found to have a moderator effect on the association between internalized stigma and adherence. The findings highlight certain trends that can inform future intervention efforts.

In our study, participants who experienced enacted stigma tended to report lower adherence, which is consistent with some previous studies conducted in Africa and South America indicating enacted stigma was a barrier to adherence while perceived stigma did not have a statistically significant association with adherence [36–40]. However, in some studies conducted in Europe and USA, perceived stigma was identified to be a barrier to adherence while enacted stigma did not have a statistically significant association with adherence [4,41,42]. The possible explanation for the finding in the current study might be that enacted stigma was related to avoidance of disclosing HIV status, which in turn led to poorer medical adherence. The mediation effect of HIV disclosure between stigma and adherence has yet to be explored through future research.

Our finding that internalized stigma was not related to adherence among the entire sample is contradictory to some previous studies which indicated that higher levels of internalized stigma were related to lower levels of adherence [17,43,44]. Some other studies, however, did not identify a statistically significant association of internalized stigma with adherence [38,42,45]. Our finding that internalized stigma was associated with adherence among participants in ethnic minority groups other than Zhuang might be explained by the synergy of stigma and ethnic minority status. HIV-positive patients who belonged to other ethnic minority groups felt further stigmatized and socially isolated, which in turn affects adherence. Future studies need to take ethnic minority groups’ cultural standards and the social context into consideration to ascertain why internalized stigma affects adherence to ART among the groups.

A positive association between age and adherence is a finding consistent with some prior studies [46,47]. In a study on adherence among older HIV patients, the association is said to be likely due to their familiarity with medication usage for chronic diseases and increased awareness that treatment of HIV requires a high level of medication adherence or due to their advanced stage of the disease that makes them take medication with a high adherence [48]. Although in general older age has been associated with better adherence, we believe that maintaining a high level of adherence will be difficult for some older PLWHA due to various challenges in their life. The explanation for the association of having a job with better adherence is that those who had a job could have more stable living conditions or afford better medications than those who did not have a job.

There are limitations to be aware of in the current study. First, the number of participants in other ethnic groups was much smaller than that in Han and Zhuang ethnic groups, which might prevent the detection of ethnicity’s moderator effects on the association between enacted or perceived stigma and adherence. Second, just two items were used to measure enacted stigma and may not truly capture the PLWHA’s experience of stigma and discrimination. Third, this study was cross-sectional in design, and hence, causal relationships between HIV-related stigma and adherence cannot be inferred. Further research is warranted to identify causality between variables employing longitudinal study designs. Fourth, some potentially important correlates such as medication regimen and clinical factors, and accessibility to opportunity of care that may influence adherence were not incorporated into analysis. Incorporating these factors into analysis in future studies may help explain the associations of stigma and ethnicity with adherence identified in the current study. For example, different ethnic groups could have different access to opportunity of care, which might be the reason for the association of internalized stigma with adherence among other ethnic minority groups. Last, data gathered through the questionnaire may be subject to both recall and social desirability biases.

In spite of the limitations, our findings demonstrate the impact of multiple types of stigma on adherence to ART and in turn health outcomes among PLWHA in Guangxi, China. Our findings also demonstrate a moderator effect of ethnicity on the association between internalized stigma and adherence. Therefore, we conclude that PLWHA of other ethnic minority groups could benefit from interventions designed to help them manage internalized stigma; meanwhile, PLWHA, overall, could benefit most from increased employment opportunities, and acquisition of coping skills to mitigate the negative effects of enacted stigma.

## Figures and Tables

**Table 1:** Adherence in relation to stigma and sociodemographic characteristics.

	Analytic sample N=2146	ART adherence group		cOR (95% CI)
		Optimal 1326 (63.3%)	Suboptimal 769 (36.7%)	
**HIV-related stigma**				
**Enacted stigma**				
** Yes**	124 (6.1%)	72 (5.5%)	52 (7.2%)	0.73^a^ (0.60, 0.94)
** No**	1894 (93.9%)	1228 (94.5%)	666 (92.8%)	
**Perceived stigma**	2.60 (0.58)	2.60 (0.57)	2.59 (0.60)	1.03 (0.90, 1.17)
**Internalized stigma**	2.31 (0.54)	2.31 (0.53)	2.32 (0.54)	0.99 (0.79, 1.24)
**Sociodemographic factors**				
**Gender**				
** Male**	1316 (61.3%)	795 (60.0%)	488 (63.5%)	
** Female**	830 (38.7%)	530 (40.0%)	281 (36.5%)	1.17 (0.96–1.43)
**Ethnicity**				
** Han**	1433 (68.5%)	901 (68.1%)	532 (69.3%)	
** Zhuang**	591 (28.3%)	379 (28.6%)	212 (27.6%)	1.10 (0.89,1.36)
** Others**	68 (3.3%)	44 (2.2%)	24 (3.1%)	1.07 (0.67,1.71)
**Local**				
** Yes**	1883 (93.5%)	1212 (93.5%)	671 (93.6%)	0.87 (0.60,1.24)
** No**	130 (6.5%)	84 (6.5%)	46 (6.4%)	
**Marital status**				
**Married/cohabitation**	1426 (69.7%)	903 (69.9%)	523 (69.4%)	1.02 (0.82,1.26)
** Other**	620 (30.3%)	389 (30.1%)	231 (30.6%)	
**Work status**				
** Full/part-time job**	1535 (73.6%)	985 (74.6%)	550 (71.8%)	1.13 (0.83,1.55)
** No job**	551 (26.4%)	335 (25.4%)	216 (28.2%)	
**Income (yuan)**				
** 0–999**	1079 (51.9%)	703 (53.4%)	376 (49.3%)	
** 1000–1999**	640 (30.8%)	394 (29.9%)	246 (32.3%)	0.90 (0.74,1.09)
** 2000–2999**	230 (11.1%)	143 (10.9%)	87 (11.4%)	0.96 (0.71,1.30)
** ≥ 3000**	129 (6.2%)	76 (5.8%)	53 (7.0%)	0.80 (0.48,1.33)
**Age**	42.37 (12.47)	43.31 (12.82)	40.72 (11.66)	1.02^a^ (1.01,1.03)
**Year of schooling**	7.01 (3.02)	6.96 (3.04)	7.11 (2.97)	0.99 (0.95,1.02)

a p<0.05, two-tailed

**Table 2:** Results of the linear regression models predicting adherence.

	Adherence		
Predictor	Model 1 aOR (95% CI)	Model 2 aOR (95% CI)	Model 3 aOR (95% CI)
**Main effect**			
Enacted stigma (yes=1)	0.68a (0.54,0.86)	0.66a (0.52,0.85)	0.53^a^ (0.33,0.87)
Perceived stigma	1.04 (0.77,1.40)	1.06 (0.76,1.49)	1.08 (0.71,1.65)
Internalized stigma	0.99 (0.66,1.49)	1.03 (0.67,1.59)	0.98 (0.58,1.65)
**Moderator**			
Ethnicity			
Han	Reference	Reference	Reference
Zhuang	1.12 (0.90,1.40)	1.08 (0.85,1.37)	0.74 (0.34,1.61)
Other	1.05 (0.65,1.69)	1.00 (0.65,1.54)	4.40 (0.71,27.29)
**Two-way interaction**			
Ethnicity*enacted stigma			
Han*enacted stigma (yes=1)			Reference
Han*enacted stigma (no=0)			Reference
Zhuang*enacted stigma (yes=1)			1.80 (0.74,4.42)
Zhuang*enacted stigma (no=0)			Reference
Other*enacted stigma (yes=1)			2.00 (0.31,12.66)
Other*enacted stigma (no=0)			Reference
Ethnicity*perceived stigma			
Han*perceived stigma			Reference
Zhuang*perceived stigma			0.90 (0.53,1.52)
Other*perceived stigma			1.31 (0.58,2.96)
Ethnicity*internalized stigma			
Han*internalized stigma			Reference
Zhuang*internalized stigma			1.30 (0.68,2.50)
Other*internalized stigma			0.40^a^ (0.17,0.96)
**Confounder variables**			
Gender (female=1)		1.16^a^ (1.05,1.29)	1.16^a^ (1.05,1.29)
Local (yes=1)		0.83 (0.59,1.18)	0.86 (0.60,1.24)
Married status (married=1)		1.02 (0.81,1.28)	1.01 (0.81,1.26)
Work status (full/part-time job=1)		1.31^a^ (1.02,1.68)	1.33^a^ (1.04,1.70)
Income			
0–999		Reference	Reference
1,000–1,999		0.91 (0.73,1.14)	0.91 (0.73,1.13)
2,000–2,999		0.92 (0.69,1.23)	0.94 (0.70,1.26)
≥ 3,000		0.77 (0.46,1.29)	0.78 (0.48,1.29)
Age		1.02^a^ (1.01,1.03)	1.02^a^ (1.01,1.03)
Year of schooling		1.02 (0.97,1.06)	1.01 (0.97,1.06)

a p<0.05, two-tailed.
